# Oriented Two‐Dimensional Porous Organic Cage Crystals

**DOI:** 10.1002/anie.201704579

**Published:** 2017-07-06

**Authors:** Shan Jiang, Qilei Song, Alan Massey, Samantha Y. Chong, Linjiang Chen, Shijing Sun, Tom Hasell, Rasmita Raval, Easan Sivaniah, Anthony K. Cheetham, Andrew I. Cooper

**Affiliations:** ^1^ Department of Chemistry Materials Innovation Factory University of Liverpool Liverpool L69 7ZD UK; ^2^ Barrer Centre Department of Chemical Engineering Imperial College London London SW7 2AZ UK; ^3^ Surface Science Research Centre Department of Chemistry University of Liverpool L69 3BX Liverpool UK; ^4^ Department of Materials Science and Metallurgy University of Cambridge Cambridge CB3 0FS UK; ^5^ Institute for Integrated Cell-Material Sciences (iCeMS) Kyoto University Kyoto 606-8501 Japan

**Keywords:** crystal defects, microporous materials, oriented molecular crystals, porous organic cages, separation membranes

## Abstract

The formation of two‐dimensional (2D) oriented porous organic cage crystals (consisting of imine‐based tetrahedral molecules) on various substrates (such as silicon wafers and glass) by solution‐processing is reported. Insight into the crystallinity, preferred orientation, and cage crystal growth was obtained by experimental and computational techniques. For the first time, structural defects in porous molecular materials were observed directly and the defect concentration could be correlated with crystal growth rate. These oriented crystals suggest potential for future applications, such as solution‐processable molecular crystalline 2D membranes for molecular separations.

Porous molecular materials are attracting much interest because they can be rationally designed to achieve functions such as selectivity, processability, and stability.^1^ For example, we have developed a series of porous organic cages (POCs) that can be used as synthetically prefabricated molecular pores for the construction of porous materials.^2^ The synthetic versatility of POCs enables a wide range of functionality and tailored properties. The porosity of crystalline cage solids arises from both intrinsic pores within the molecules themselves and extrinsic pores between the cages. The packing of discrete cage molecules is dictated by weak van der Waals forces that give scope for dynamic motion, flexibility, and response to stimuli.^3^ Also, unlike covalent organic frameworks (COFs), POCs are crystallized without any bond‐forming reactions; hence, while single‐crystalline COFs are very rare,^4^ it is relatively easy to grow high‐quality single‐crystal POCs. POCs have been explored in various applications such as sensing,^5^ gas storage,^2^ molecular separations (for example, xylene isomers,^6^ noble gases,^7^ and chiral molecules^8^), and proton conductivity.^9^ As discrete, soluble molecules, POCs can be processed in organic solvents in a way that cannot be achieved with insoluble porous frameworks. For example, modular mix and match assembly strategies have been used to form binary and ternary cocrystals,^10^ and cage crystals can be incorporated into polymers to form composite membranes.^11^

The fabrication of functional materials into thin films, membranes, and oriented crystals on substrates is of importance for applications in sensors, catalysts, electronic devices, and electrodes for fuel cells.^12^ Recently, amorphous cage thin films and membranes were fabricated on various substrates by spin coating.^13^ Uniform and pinhole‐free cage membranes were obtained and demonstrated molecular‐sieving properties. However, it remains a significant challenge to control crystallinity, orientation, and surface nanostructures of cage thin films; for example, these amorphous spin‐coated films showed dramatic ageing effects over time. As such, the preparation of crystalline POC films is a high‐value target for applications such as gas separation. Various studies have been carried out on the assembly of well‐organized 2D molecular systems, such as growth and alignment of organic semiconductor thin films.^14^ Likewise, porous frameworks such as zeolites and metal–organic frameworks (MOFs) have been fabricated into thin films and membranes.^15^ However, there are fewer studies of crystalline oriented MOFs or zeolite films.^16^ Most of the films are polycrystalline, and well‐controlled growth and orientation is challenging. Porous thin‐film materials with high crystallinity and preferred orientation should present distinct adsorption, separation kinetics, and performance characteristics compared with bulk powders or amorphous films.

Orientation is not the only factor that affects guest diffusion in porous solids: defect engineering in porous frameworks has emerged as an active research field because defects can play a vital role in determining material performance such as sorption capacity, catalytic activity, stability, and mechanical strength.^17^ However, our ability to characterize, understand, and control defects in porous solids is limited.^18^ The presence of defects in MOFs may explain oft‐noted discrepancies between properties derived from ideal crystal structures and experimental measurements. POCs are interesting systems for investigating defects because, unlike MOFs and COFs, the synthesis and the crystallization steps can be separated. The formation of defects such as point vacancies is thermodynamically unfavorable,^19^ and it would be expected to be rare in molecular crystals. However, there is indirect evidence for the existence of defects in POCs;^20^ for example, when cage molecules were crystallized both slowly and rapidly, the rapidly crystallized sample exhibited substantially higher surface areas, despite both samples showing similar powder diffraction patterns.^21^ Rapid crystallization would be expected to give crystals with more defects resulting in more extrinsic porosity and higher gas uptakes.21a It is challenging to characterize structural defects for bulk polycrystalline powders, and until now, defects have not been observed directly in POCs.

Herein, we report the oriented assembly of POC crystals on surfaces such as silicon wafers and glass substrates. Cage crystals were grown as 2D hexagonal layers, aligned in parallel with the substrate. This new morphology was fabricated by the simple technique of dip coating. Local point defects were directly observed, for the first time, using atomic‐force microscopy (AFM). A “perfect” aligned cage crystal was obtained using a slow crystallization process, while molecular vacancies were formed by rapid removal of the solvents. The concentration of defects was also found to be related to the crystal growth rate.

CC3 was synthesized as described previously.^2^ This cage molecule has tetrahedral symmetry with an internal void and four open triangular windows (Figure 1 A,B). The crystal structure, CC3α, shows a window‐to‐window packing motif, leading to an interconnected 3‐dimensional (3D) pore channel, as shown in Figure 1 D–F.^22^ The Brunauer–Emmett–Teller (BET) surface area of CC3α is 409 m^2^ g^−1^ when it forms a highly crystalline solid.21a

**Figure 1:**
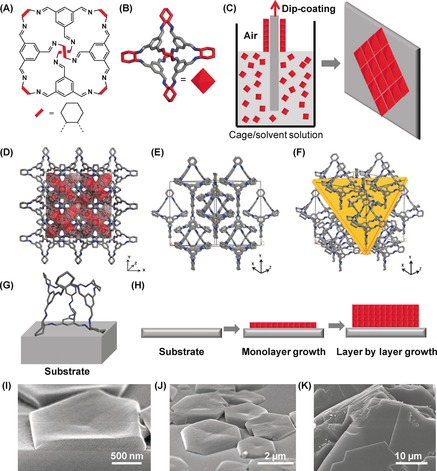
A) Molecular structure of CC3. B) 3D structure of CC3 showing tetrahedral symmetry. C) Representation of controlled dip coating of CC3 on a substrate to form oriented layers. D) Crystal structure of CC3 with a Connolly surface in red viewed along the *y* axis. E) Crystal structure of CC3 along the *xz* orientation. F) (111) plane in CC3 crystal structure, displayed in yellow. G) Noncovalent intermolecular interaction between the cage molecule and solid substrate. H) Representation of layer by layer growth of cage molecules on a substrate. I) Cross‐sectional SEM view of one oriented cage CC3 crystal on a silicon wafer substrate. J) SEM image for oriented CC3 crystals grown on a silicon wafer. K) Cross‐sectional SEM image of a bulk oriented CC3 crystal showing multiple layers of molecular cage sheets.

We developed a simple and efficient method to create oriented CC3 structures on substrates by dip coating. As illustrated in Figure 1 C and the Supporting Information, Figure S1, the substrate was immersed into a solution of CC3 in chloroform or dichloromethane for an appropriate period of time to grow oriented seed crystals. By pulling the substrate upward at a constant speed, oriented cage crystals were formed on the substrate upon solvent evaporation. The cage molecules preferentially nucleate and adhere to the surface of the substrate via van der Waals interactions, and are subsequently assembled into aligned crystalline layers or films (Figure 1 G,H).

Scanning electron microscopy (SEM) images revealed that cage molecules formed hexagonal shaped crystals on the surface of a silicon wafer (Figure 1 I,J), in contrast to the octahedral morphology of bulk CC3 crystals (Figure 2, insets; Supporting Information, Figure S2). The diameter of the hexagonal shaped crystals was 3–5 μm with an average thickness of about 200 nm, and these microcrystals were formed discontinuously on the substrate (that is, the substrate was not fully covered; Supporting Information, Figure S3). These hexagonal cage crystals could also be fabricated on other substrates such as glass and carbon TEM grids (Supporting Information, Figures S4,S5).

**Figure 2:**
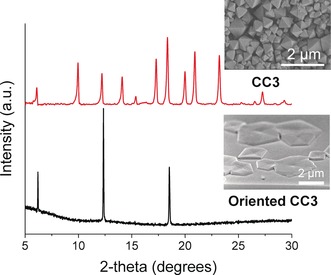
PXRD patterns of bulk 3D CC3 crystals (upper) and oriented 2D CC3 crystals grown on silicon wafer (lower). SEM images show octahedral crystals for bulk CC3 and hexagonal shaped crystals of oriented CC3.

The crystallinity and preferred orientation of these aligned CC3 crystals were further characterized by powder X‐ray diffraction (PXRD). The PXRD patterns of oriented CC3 crystals fabricated on the surface of silicon wafer show that they possess cubic *F*4_1_32 symmetry with *a*=25.4 Å, in good agreement with bulk CC3 crystals (Figure 2; Supporting Information, Figure S7). Only three diffraction peaks are observed for the surface deposited CC3 crystals, indicating the oriented nature of the materials. The peaks can be indexed as (111), (222), and (333). Therefore, CC3 cage molecules were grown in the (111) direction on the silicon wafer surface. The cage packing along (111) orientation is illustrated in the Supporting Information, Figure S8. AFM was also used to characterize these oriented crystals. Figure 3 A shows the AFM image of an entire hexagonal shape cage crystal grown on a silicon wafer substrate. Figure 3 C,D show the individual terraces on top of the cage crystals. The height of these terraces is measured as 1.41±0.18 nm, which agrees well with the size of cage molecule as measured from the single crystal structure. A topographic study of oriented CC3 crystals (Figure 3 E) shows the cage packing structure on the crystal surface. The height profile showed that the cage molecules have an intermolecular spacing of 1.41±0.18 nm (Figure 3 F). Both PXRD analysis and AFM images suggest that the cage molecules are assembled by a layer‐by‐layer growth mechanism with preferential (111) orientation.

**Figure 3:**
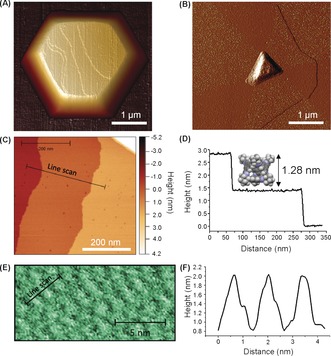
AFM analyses of surfaces of oriented cage crystals. A) AFM image of a defect‐free oriented cage crystal on silicon wafer. B) AFM PeakForce error image of a quickly‐grown oriented CC3 crystal showing the segments of the hexagonal crystal. C),D) Line scan of individual terrace steps from (B) on top of the crystals showing a step height of 1.41±0.18 nm, with a space‐filling model of CC3 shown to scale of the *y* axis. E) AFM topographic image of a well‐grown oriented cage CC3 crystal. F) Height profile along the pathway as shown in the image of (E).

Precise control of the dip‐coating method (see the Supporting Information) allowed us to adjust the nanostructure of the crystals to produce either near‐perfect crystals with very few defects or crystals with a high number of vacancy defects. AFM showed that the local morphology was affected by the growth conditions. High‐resolution AFM deflection imaging of a slowly crystalized sample showed a flat, hexagonal shape and defect‐free crystal surfaces (Figure 3 A; Supporting Information, Figure S9). We also prepared quickly grown oriented CC3 crystals. AFM images revealed a hexagonal crystal with a triangular nucleation point in the center (Figure 3 B; Supporting Information, Figure S10) surrounded by six segments relating to the hexagonal packing of cages in the crystal structure. Molecular vacancies were observed on the surface of the crystal, as shown in Figure 3 B and Figure 4. The defects on the crystal surface are localized within three of the six segments (Figure 3 B, and Figure 4), producing an alternating pattern of high and low defect concentration. More AFM images of other quickly grown CC3 crystals also show a similar pattern and a large number of vacancy defects (Supporting Information, Figures S11,S12). This pattern is related to the growth of the segments and the crystallographic directions (Supporting Information, Figure S13). The segments of the crystal formed at the apex of the central triangular defect have 2 % surface vacancies while the segments formed at the edges of the triangle have 10–12 % surface vacancies. Initial formation of a triangular {111} face by growth of the crystal parallel to the surface, followed by propagation from the vertices (parallel to ⟨100⟩) and edges (along ⟨110⟩) would account for the observed crystal shape. Differences in vacancies that is, “missing cages”, and void defect concentrations between the sectors can be related to edge versus point growth, with the probability of imperfections higher for growth from the edge, owing to the larger area of the growth front and potential differences in the both intermolecular interactions presented by the cages in this direction. The size of the defects ranges from individual molecular vacancies up to 27.5 nm multiple vacancy pores, which indicates that multiple cage molecules are absent during the rapid crystal growth. Furthermore, Figures 3 B and S10 show that the defects are not just present at the surface. The formation of a new layer of cage molecules growing on top of the crystal surface with defects beneath it suggests that additional pore volume owing to defects is retained in the subsurface crystal structure. This is the first direct evidence for the existence of vacancy defects, which have been invoked previously to explain the properties of porous molecular materials.^20^, ^21^ This explains our observation, for example, that rapidly crystallized bulk CC3 has significantly higher surface area than slowly crystallized CC3.

**Figure 4:**
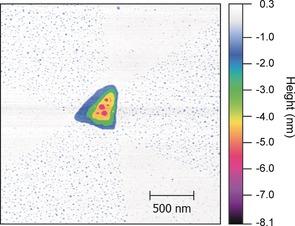
An AFM image showing the central triangular defect of a cage crystal which was grown with defects (2.5×2.5 micrometer scan). The individual layers of the crystal can be seen, the top layer is white, 2nd blue, 3rd green, 4th yellow, and the bottom layer is magenta. The three hyperporous areas show an increase in blue speckled areas corresponding to molecule vacancies in the top surface layer.

The ability to control and to quantify vacancies as a direct function of crystallization rate demonstrates a viable “defect engineering” strategy for POCs via controlled solution processing.

We also tried to grow oriented cage films on glass substrates. Microscopy showed large hexagonal crystals grown continuously on the glass surface (Supporting Information, Figure S14), suggesting potential for forming conformal porous crystalline coatings. The key to successful growth of these uniformly oriented large crystals was appropriate solvent evaporation conditions. The resultant bulk oriented crystals exhibited multiple cage layers (Figure 1 K; Supporting Information, Figure S14). PXRD shows three main peaks at 2*θ*=6.2° (111), 12.4° (222), and 18.6° (333) (Supporting Information, Figure S15), indicating that oriented growth on glass occurs parallel to the (111) crystal planes, as for the silicon surface. After the oriented crystals were ground to fine powders, the PXRD was fully consistent with the known CC3 crystal structure^2^ (Supporting Information, Figure S16). Hence, the oriented CC3 crystals pack window‐to‐window, but grow in a preferentially oriented manner. In addition, a multiple dip‐coating process was carried out to promote secondary growth of oriented cage crystals. After more than 100 cycles of dip coating, the substrate was densely covered by discrete hexagonal crystals with a surface coverage of up to 85 %, although the orientation was lost on the uppermost layers (Supporting Information, Figure S17).

Simulations were used to generate representative structural models of the interactions between CC3 and the silicon surface. There are two possible geometries for the growth of oriented cage crystals on silicon, with either the cage window or cage arene face attached to the surface. The atomistic model for each of these cases (Figure 5 A–D) was geometry‐optimized at the PBE‐D3 level of theory, using the CP2K package.^23^ Surface binding energies derived from these models showed that the cage arene interacts with the silicon wafer more strongly by 16.1 kJ mol^−1^. The structural model of oriented cage crystals (Figure 5 E) was constructed from a starting model based on the reported crystal structure of CC3α, with 2D layers of cage molecules grown in the (111) direction.

**Figure 5:**
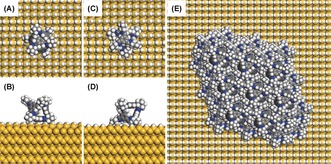
Atomistic models of cage molecules on the surface of silicon (100) wafer. A single cage molecule can sit on the silicon surface with a cage arene face (A, B; two viewing directions) or a cage window (C, D) attached to the surface. E) Structural model of oriented cage crystals. The cage molecules are assembled in a window to window packing arrangement with a preferred orientation as refined from PXRD.

In summary, this study demonstrates the controlled surface growth of aligned cage crystals for the first time. Cage molecules can be grown in a preferentially oriented manner on several substrates. A structural model was generated to represent the cage packing motifs on a silicon substrate. The dip‐coating approach is a simple and efficient way to fabricate porous molecular materials into thin films with control over defect concentration. This is the first time that defects have been observed directly in crystalline POCs, and the defect concentration can be correlated with the crystallization rate. These results suggest new opportunities for these molecular cage materials; for example, large coherent crystalline POCs thin films might be useful for molecular sieving, allowing the excellent potential that has been demonstrated for bulk POCs^7^, ^24^ to be transferred into more practicable and scalable membrane technologies.

## Conflict of interest

The authors declare no conflict of interest.

## Supporting information

As a service to our authors and readers, this journal provides supporting information supplied by the authors. Such materials are peer reviewed and may be re‐organized for online delivery, but are not copy‐edited or typeset. Technical support issues arising from supporting information (other than missing files) should be addressed to the authors.

Supplementary
