# Supplementary material for: Oriented Two‐Dimensional Porous Organic Cage Crystals
Source: Angew Chem Int Ed Engl. 2017 Jul 6;56(32):9391–5. doi: 10.1002/anie.201704579 (PMC5577517; doi:10.1002/anie.201704579)
Supplement: Supplementary file 1 — Supplementary [file ANIE-56-9391-s001.pdf]

Angewandte Chemie
Eine Zeitschrift der Gesellschaft Deutscher Chemiker

Supporting Information

Oriented Two-Dimensional Porous Organic Cage Crystals

Shan Jiang, Qilei Song, Alan Massey, Samantha Y. Chong, Linjiang Chen, Shijing Sun, Tom Hasell,* Rasmita Raval, Easan Sivaniah, Anthony K. Cheetham, and Andrew I. Cooper**

anie_201704579_sm_miscellaneous_information.pdf

Author Contributions

S.J. Data curation: Equal; Formal analysis: Equal; Investigation: Equal; Methodology: Equal; Writing—original draft: Lead; Writing—review & editing: Supporting
Q.S. Conceptualization: Lead; Data curation: Equal; Investigation: Equal; Methodology: Equal; Writing—review & editing: Lead
A.M. Data curation: Supporting
A.C. Funding acquisition: Lead; Project administration: Lead; Resources: Lead; Supervision: Equal; Writing—review & editing: Lead.

Supporting Information

Table of contents

1. **Experimental**
2. **Methods**
3. **Characterizations**

Figure S1. Schematic diagram of formation of crystalline oriented **CC3** crystals on substrates by controlled evaporation and dip-coating.
Figure S2. SEM images of octahedral **CC3** crystals grown in solution.
Figure S3. Scanning electron microscopy (SEM) images of oriented cage crystals grown on the silicon wafer.
Figure S4. Oriented **CC3** crystals on glass substrate.
Figure S5. Oriented cage crystals grown on the carbon grid.
Figure S6. Amorphous **CC3** thin films coated on silicon wafer. The samples were prepared by manual dip-coating of **CC3** cage solution.
Figure S7. PXRD patterns for bulk **CC3** crystals and oriented **CC3** crystals deposited on silicon wafer.
Figure S8. The cubic **CC3** structure viewed along the [111] direction, showing hexagonal arrays of cage molecules arranged in layers parallel to the (111) plane.
Figure S9. AFM images of 2D **CC3** crystals on silicon wafer.
Figure S10. 5 x 5 micron AFM image of the central defect of a quickly-grown **CC3** crystal 3D topographic image.
Figure S11. Optical microscope image of crystals grown for AFM.
Figure S12. AFM images of additional **CC3** crystals with defects.
Figure S13. Schematic showing the relationship between the morphology of **CC3** crystals and the underlying cubic crystal structure.
Figure S14. Bulk oriented cage crystals grown in the glass vial.
Figure S15. Final observed (red circles), calculated (black line) and difference (blue line) transmission PXRD profiles for Le Bail refinement of bulk oriented **CC3** crystals grown in the sample vial.
Figure S16. Final observed (red circles), calculated (black line) and difference (blue line) PXRD profiles for Le Bail refinement of bulk oriented **CC3** crystals after grinding.
Figure S17. SEM images of oriented cage crystals on silicon wafer prepared by multiple dip coating process.

1. Experimental

Materials: 1, 3, 5-Triformylbenzene was purchased from Manchester Organics, (1R, 2R) - 1, 2-diaminocyclohexane was purchased from TCI-UK. Silicon (100) wafer were purchased from University Wafer US. All other chemicals were purchased from Sigma Aldrich and used as received.

Synthesis of CC3:

100 ml dichloromethane (DCM) was added into 1, 3, 5-triformaylbenzene (1.0 g, 6.2 mmol). A solution of (1R, 2R) - 1, 2-diaminocyclohexane (1.1 g, 9.3 mmol) in 100 ml DCM was added. The mixed solution was covered and left in the room temperature for 5 days. The crystalline product was obtained by solvent evaporation and washed with 95% methanol (MeOH)/5% DCM. Yield: 85%.

Fabrication of oriented cage crystals on silicon wafer substrate by dip coating:

CC3 was dissolved in chloroform ($CHCl_3$) with a concentration of 5 mg/ml. The sample vial was left open to air during the dip coating process. A program has been set up as follows to prepare perfect 2D aligned cage crystals: the silicon wafer substrate (approximately 1 cm x 3 cm) was immersed into the cage solution at a constant of dipping speed of 2 mm/min followed by a dwell time of 15 min. The substrate was then pulled upward at a constant withdrawal speed of 2 mm/min followed by a drying time of 15 min in air. The travel distance when pulling up or down was 25 mm. 20 dip coating cycles were used to achieve a good surface coverage.

For the 2D aligned cage crystals with defects: the substrate (approximately 1 cm x 3 cm) was immersed into the cage solution at a constant of dipping speed of 100 mm/min followed by a dwell time of 15 min. The substrate was then pulled upward at a constant withdrawal speed of 100 mm/min followed by a drying time of 15 min in air. The travel distance when pulling up or down was 25 mm. The substrate was dipped with 20 cycles.

A manual dip-coating process has been used to prepare 2D aligned cage crystals. The silicon wafer substrate was immersed into the **CC3** $CHCl_3$ solution and then was pulled out by hand. However, amorphous cage films could be formed due to rapid and uncontrollable solvent evaporation, as shown in Figure S6 and S7 (b).

Growth of large oriented cage crystals:

60 mg **CC3** was dissolved in co-solvent of 2 ml DCM and 0.1 ml MeOH. The cage solution was kept in the glass vial and left for a slow solvent evaporation. Hexagonal shaped crystals were grown on the internal surface of glass vial after 30 days as shown in Figure S14 (a-b).

2. Methods

Optical Microscopy: Imaging was achieved using a Meiji Techno MX9300 optical microscope fitted with an Infinity1 digital camera. Images were collected using either 4x, 10x or 25x magnification lenses. Images were processed using Infinity Analyze software 4.6.0 (Lumenera Corporation) calibrated against a stage micrometer (Agar Scientific).

Scanning Electron Microscopy: High resolution imaging of the crystal morphology was achieved using a Hitachi S-5500 cold Field Emission Scanning Electron Microscope (FE-SEM). The dry samples were prepared on 15 mm Hitachi M4 aluminium stubs using either silver dag or an adhesive high purity carbon tab. The samples were then coated with a 2 nm layer of gold using an Emitech K550X automated sputter coater. The FE-SEM measurement scale bar was calibrated using certified SIRA calibration standards. Imaging was conducted at a working distance of 8 mm and a working voltage of 3 kV using a mix of upper and lower secondary electron detectors.

Powder X-ray Diffraction:

Powder X-ray diffraction data for oriented cage crystals on silicon wafer substrate were recorded on a Bruker D8 X-ray diffraction machine with Cu Kα radiation (λ = 1.5418 Å) in the range of 5 – 40° to examine the crystallinity of bulk **CC3** crystals and oriented **CC3** crystals grown on silicon wafer. A step size of 0.01° with 0.6 s per step was employed.

XRD data for large oriented cage crystals grown on the glass surface were collected on a Panalytical X'pert pro multi-purpose diffractometer in transmission geometry operating with a Cu anode at 40 kV 40 mA. The loose crystals were mounted on thin Mylar film held in an aluminium well plate orthogonal to the incident beam and XRD patterns were collected with a step size of 0.013º 2θ and scan time of 248 s/step over 5–50º 2θ using a PIXcel detector operating in scanning mode. PXRD data were collected for the same crystals after grinding by pestle and mortar contained in a 0.7 mm borosilicate glass capillary in transmission geometry on a Panalytical Empyrean diffractometer producing Cu Kα radiation and equipped with an X-ray focussing mirror. Data were collected using a PIXcel 3D detector in 1D scanning mode. Le Bail fitting was performed using *TOPAS-Academic*.[1]

Atomic force microscopy: The Si substrate samples were mounted on a magnetic 12 mm diameter puck. All AFM images were taken in air using PeakForce tapping mode on a Multimode 8 AFM (Bruker). The images were acquired using Scanasyst Air and Fluid+ probes

(Bruker) with a nominal spring constant of 0.4 and 0.7 N/m respectively. Images were processed using both NanoScope Analysis 1.5 software (Bruker) and Gwyddion.[2]

Molecular Simulation:

We computationally evaluated the binding of a single cage molecule onto the (100) surface of silicon. A 2-dimensional slab of silicon was carved out of the crystal structure, with all dangling bonds on both surfaces terminated by dihydrides. The slab was surrounded by a vacuum region on either side. The surface binding energy was given by:

E_{binding} = *E*(silicon slab•••cage molecule) – *E*(silicon slab) – *E*(cage molecule)

where *E*(silicon slab•••cage molecule), *E*(silicon slab) and *E*(cage molecule) are the total energies of the silicon slab with a cage molecule adsorbed on the surface, the silicon slab on its own, and the isolated cage molecule, respectively.

The silicon slab was placed in an orthorhombic simulation box, which had the dimensions of 5.8 nm × 5.8 nm × 7.2 nm and was repeated infinitely in three dimensions. The slab was in parallel to the XY plane (replicated in the X and Y directions) and had a thickness of 2.2 nm in the Z direction; the slab and its next periodic image in the Z direction was separated by a vacuum region with a thickness of 5 nm. This slab model was geometry-optimized, allowing both atom positions and box lengths to relax while maintaining the orthorhombic box shape. The geometry optimization was first done with density based tight binding (DFTB) methods, followed by a further relaxation using Perdew–Burke–Ernzerhof (PBE) exchange–correlation functional with semi-empirical dispersion corrections from the DFT-D3 method.

With this fully optimized silicon slab, we generated substrate-adsorbate configurations for a single cage molecule on the silicon slab surface, using the Adsorption Locator module in BIOVIA Materials Studio 2017, in conjunction with the COMPASS II force field. The most stable surface adsorption configuration of the cage was found to be having one of its arene faces attached to the silicon surface. This substrate-adsorbate configuration was further geometry-optimized, using DFTB and then PBE-D3, as described above. For comparison, the most stable configuration where one of the cage windows was attached to the silicon surface was also selected and fully optimized, using DFTB and then PBE-D3.

To determine *E*(cage molecule), a single cage molecule was placed at the centre of an otherwise empty simulation box, having the dimensions of 5.8 nm × 5.8 nm × 7.2 nm. The cage molecule was fully relaxed, using PBE-D3, while keeping the simulation box fixed. All surface binding energies were determined at the PBE-D3 level of theory, based on the geometries optimized at the same level.

All DFTB and DFT simulations were performed with the CP2K package. DFTB in the self-consistent-charge parameterization was used in combination with a UFF dispersion correction. All PBE-D3 calculations made use of the MOLOPT basis sets of the double-ζ quality, together with the Goedecker–Teter–Hutter pseudopotentials. The charge-density cutoff for the auxiliary plane-wave expansions was set to 350 Ry.

3. Characterizations

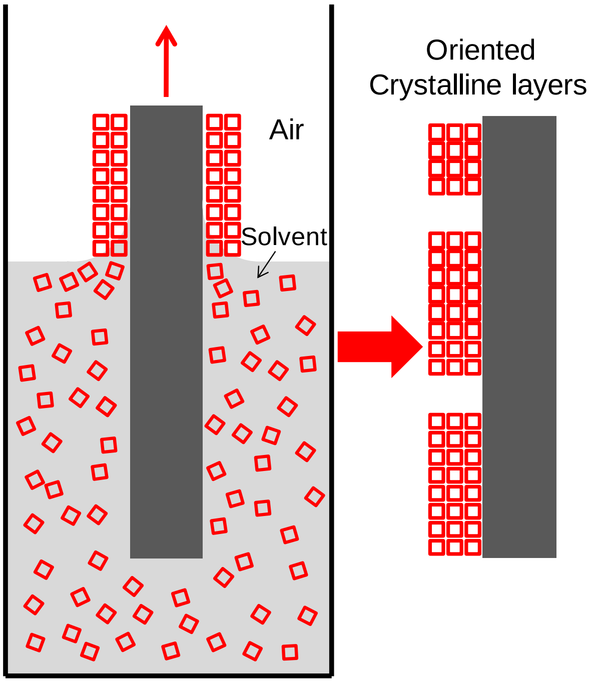

Figure S1. Schematic diagram of formation of crystalline oriented **CC3** crystals on substrates by controlled evaporation and dip-coating. Dip-coating of **CC3** to form oriented crystals on substrate, by slowly pulling the substrate out the solution with the solvent evaporated in the air, resulting in cage molecules assembled on the surface in order.

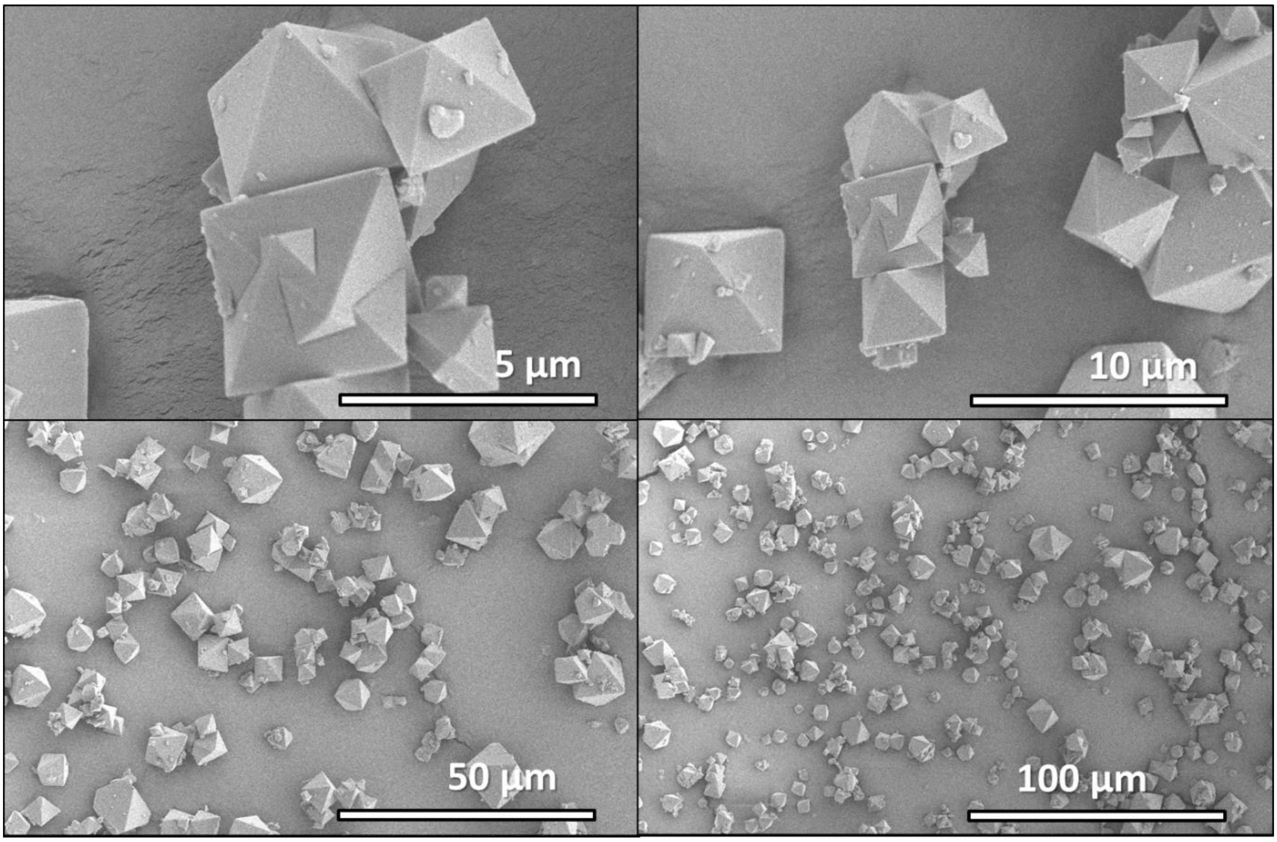

Figure S2. SEM images of octahedral **CC3** crystals grown in solution.

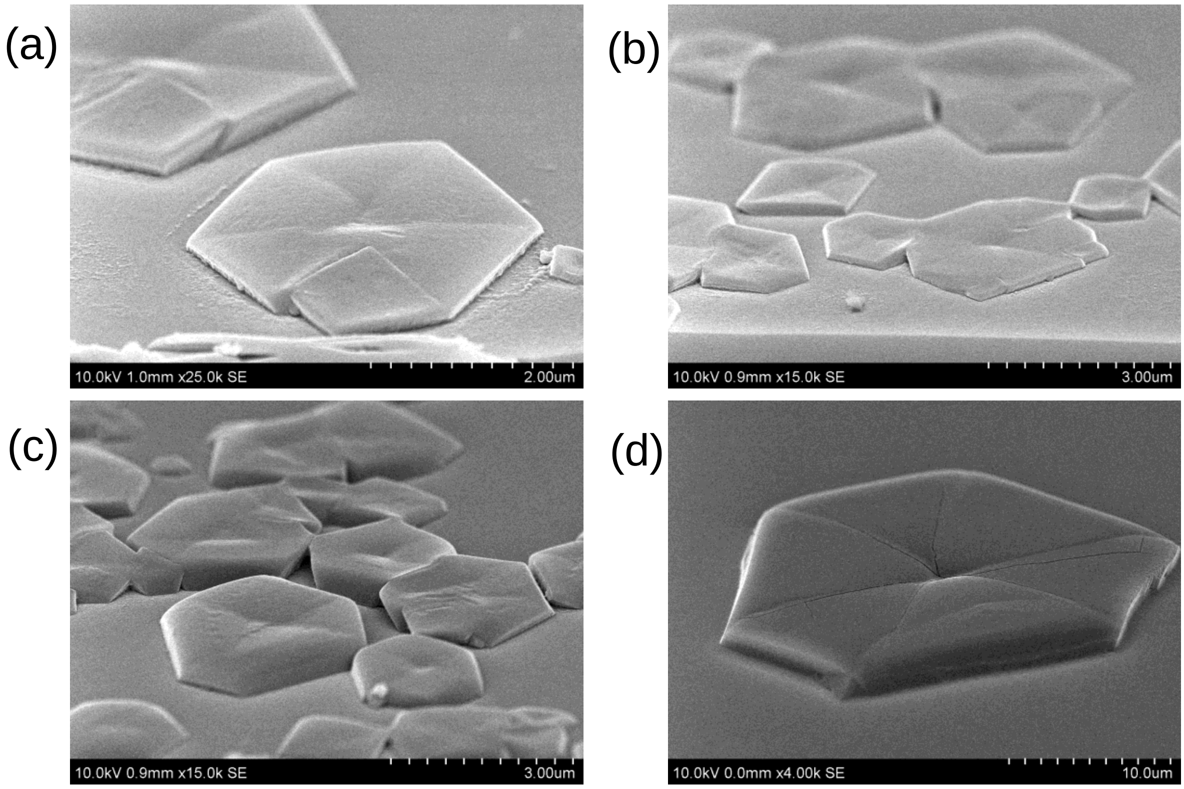

Figure S3. Scanning electron microscopy (SEM) images of oriented cage crystals grown on the silicon wafer.

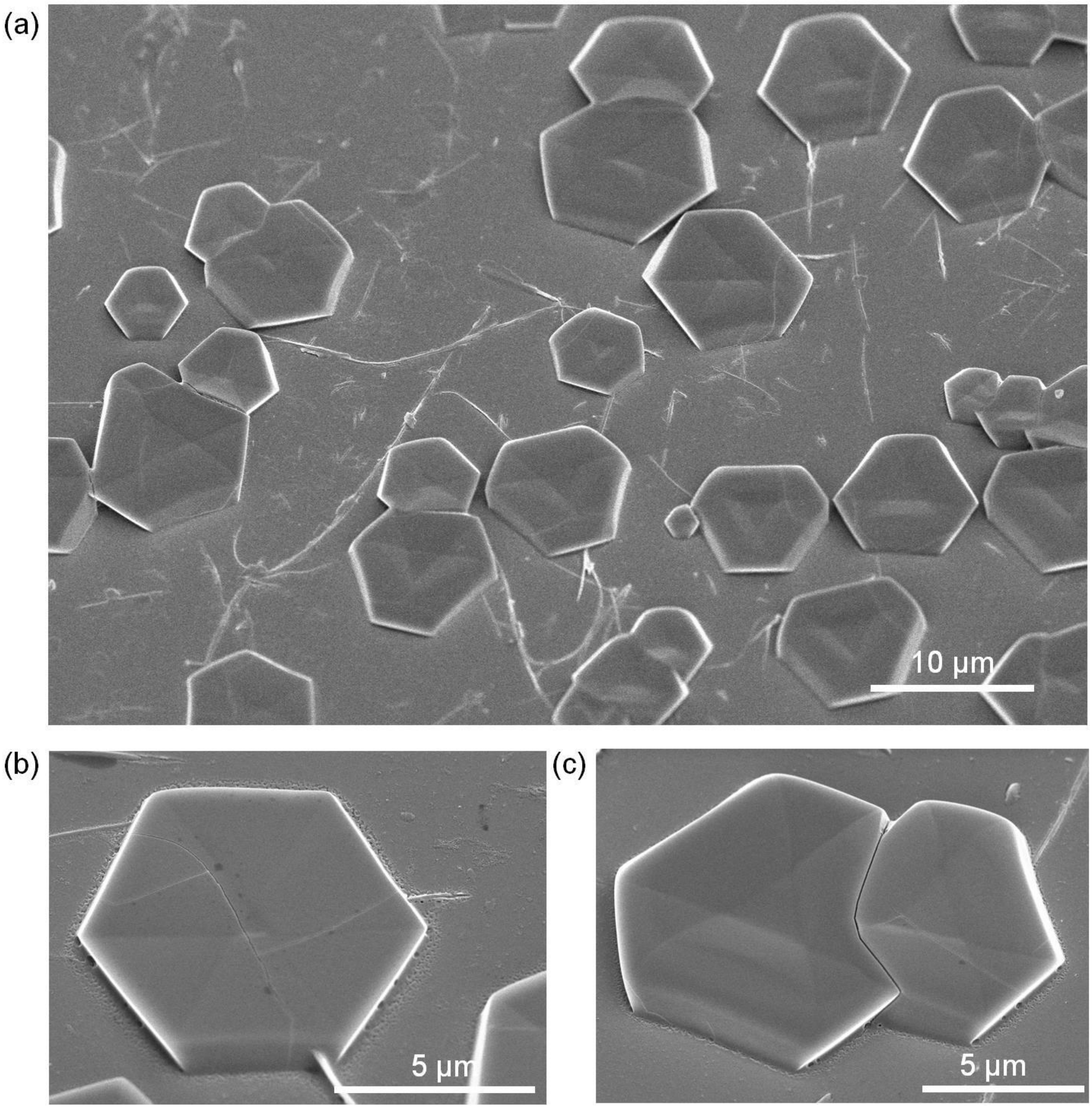

Figure S4. Oriented **CC3** crystals on glass substrate.

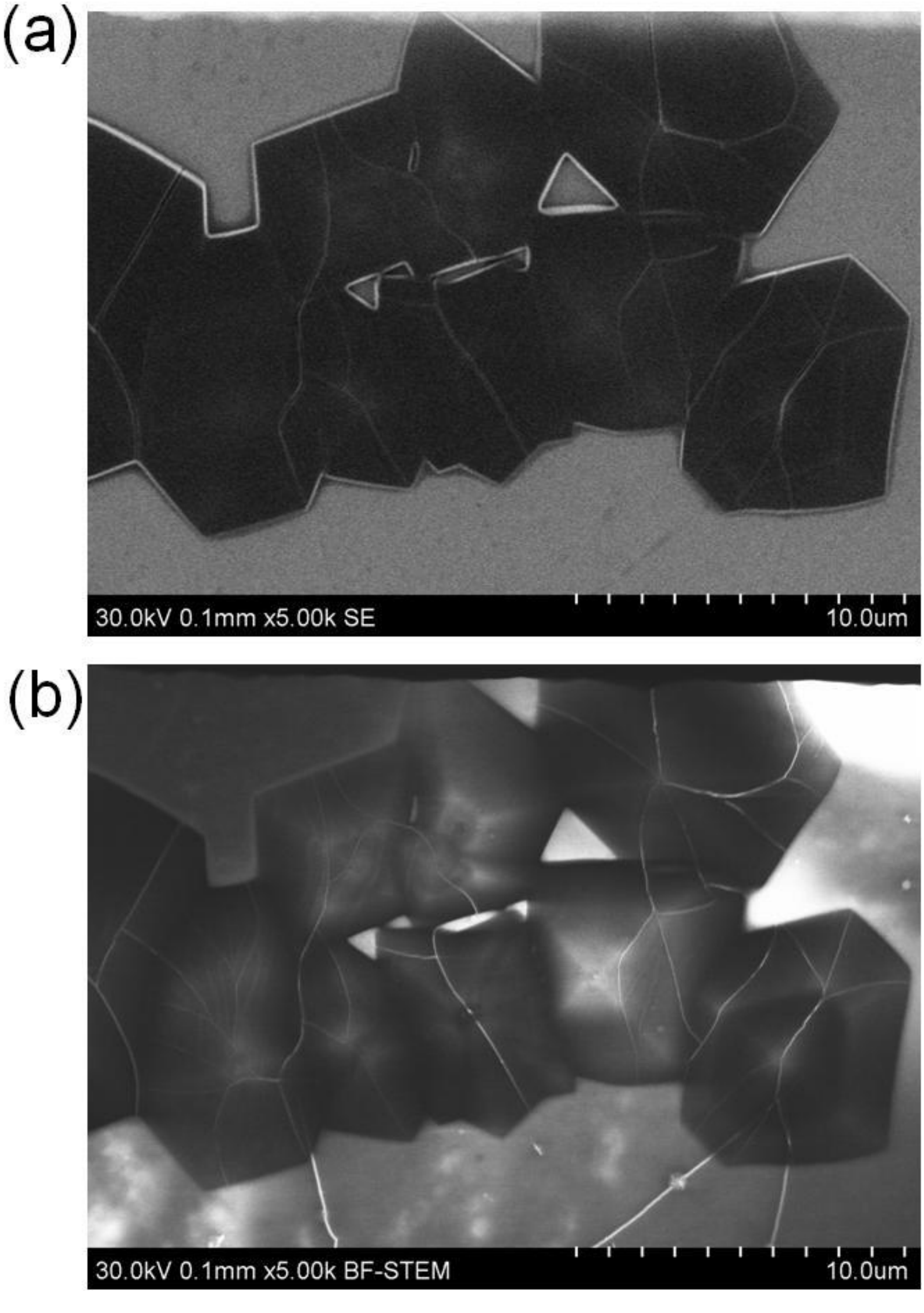

Figure S5. Oriented cage crystals grown on the carbon grid. The samples were prepared by dip-coating of **CC3** cage molecules on TEM grid. (a) SEM of 2D **CC3** crystals on carbon films, (b) Bright-field STEM image showing **CC3** crystals.

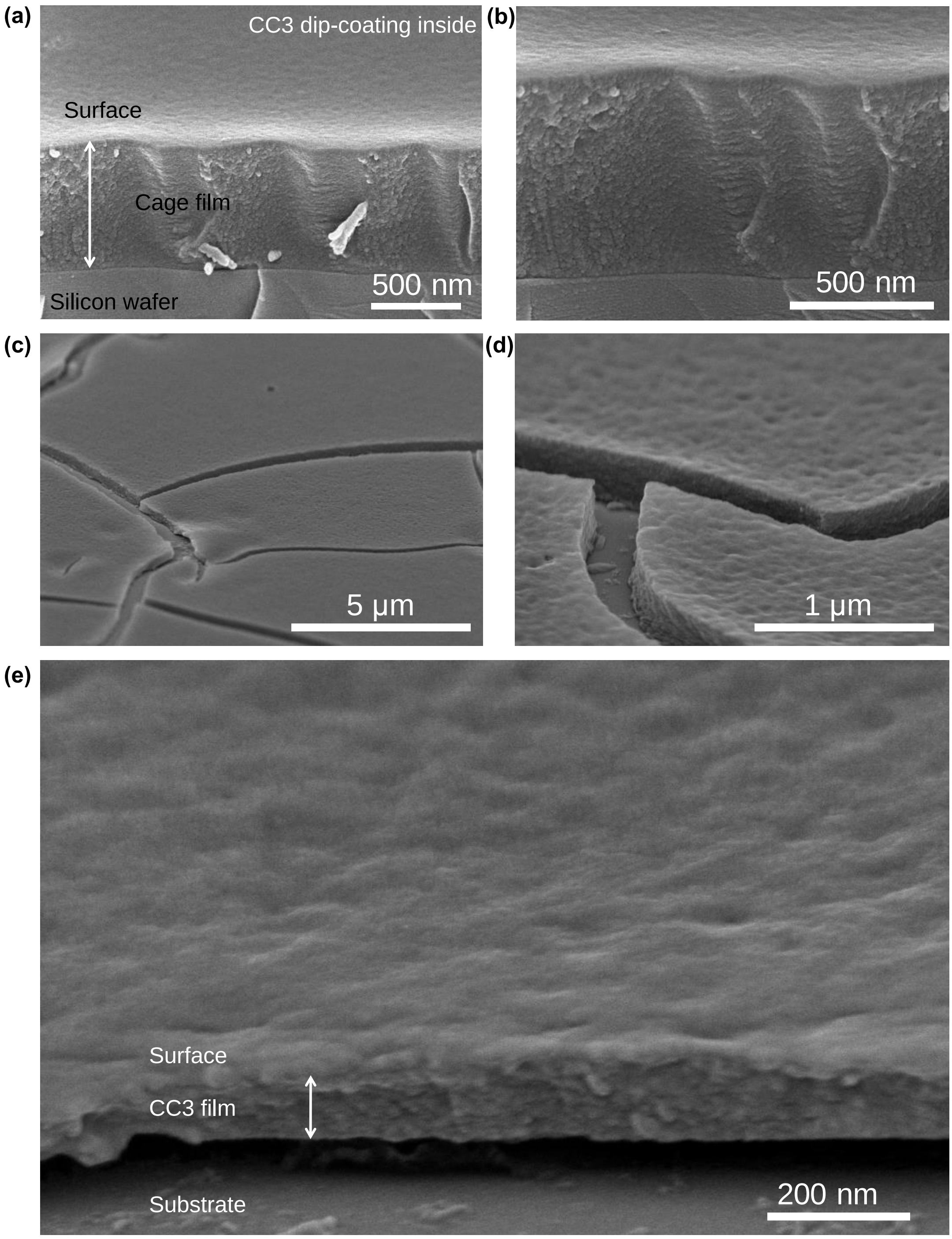

Figure S6. Amorphous **CC3** thin films coated on silicon wafer. The samples were prepared by manual dip-coating of **CC3** cage solution.

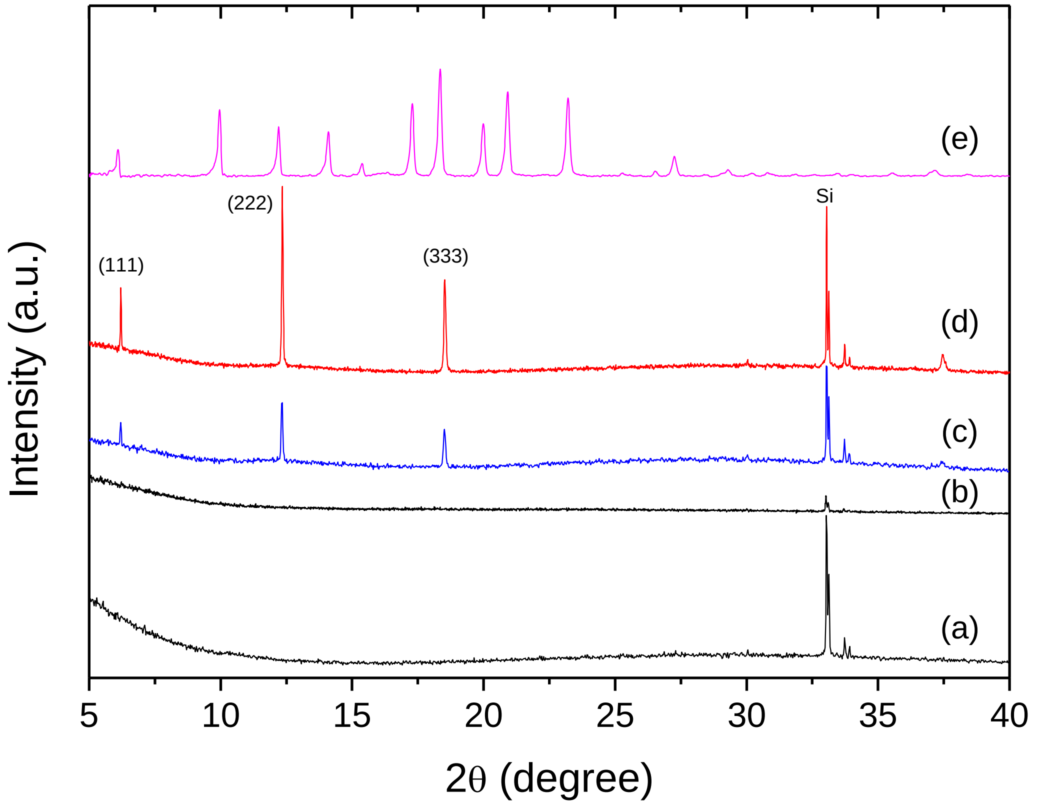

Figure S7. PXRD patterns for bulk **CC3** crystals and oriented **CC3** crystals deposited on silicon wafer. (a) Silicon wafer substrate, (b) amorphous **CC3** dip-coated on silicon wafer (Figure S6), (c) oriented **CC3** crystals dip-coated on silicon wafer, (d) oriented **CC3** crystals on silicon wafer, (e) bulk **CC3** crystals.

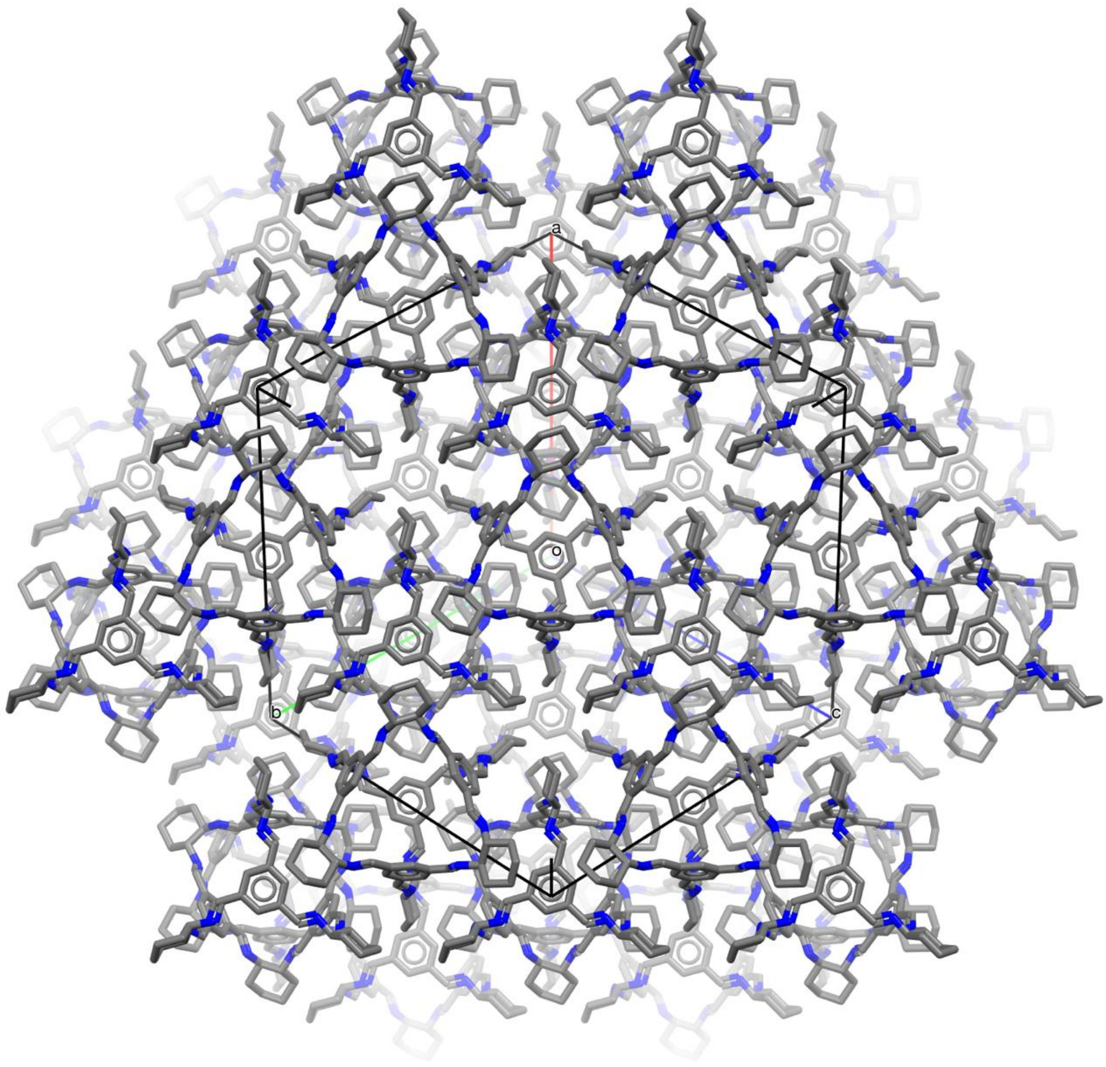

Figure S8. The cubic **CC3** structure viewed along the [111] direction, showing hexagonal arrays of cage molecules arranged in layers parallel to the (111) plane.

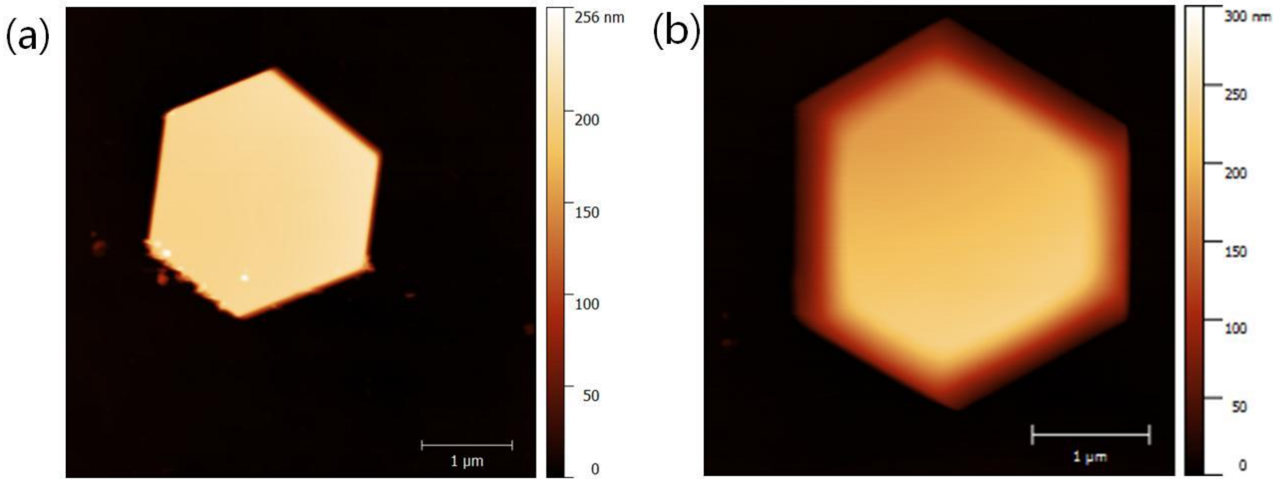

Figure S9. AFM images of 2D **CC3** crystals grown defect-free on a silicon wafer.

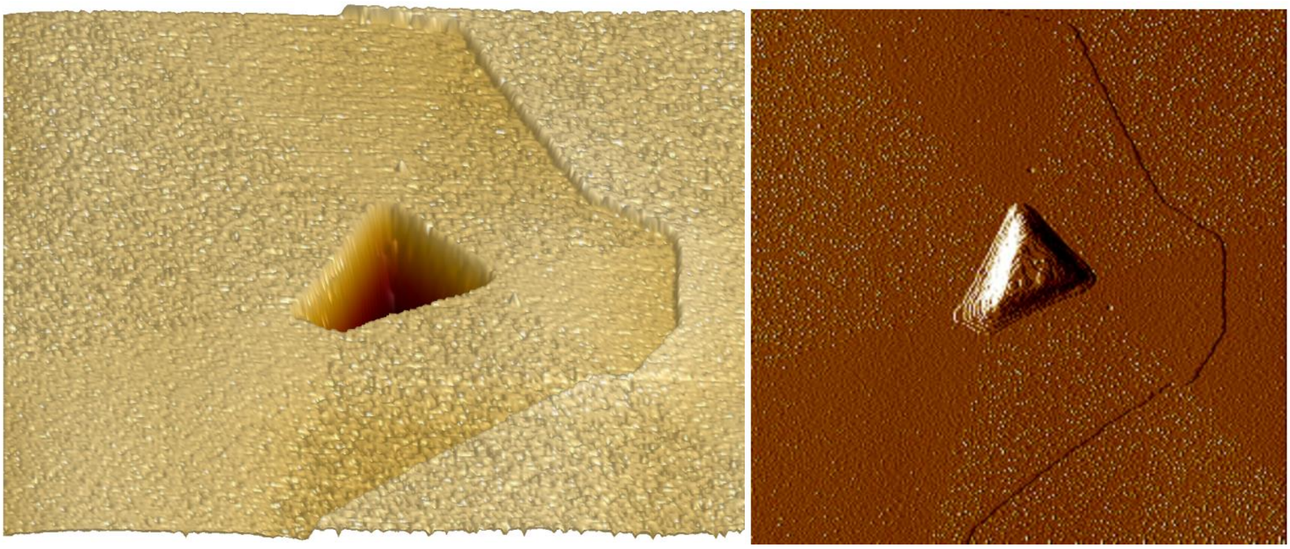

Figure S10. 5 x 5 micron AFM image of the central defect of a quickly-grown **CC3** crystal 3D topographic image showing an new layer propagating from the right side if the image on top of defect rich areas of the crystal and the PeakForce Error channel of the same data showing the concentration of defects with a 3-pointed star motif. This is a different crystal to that shown in Figure 3.

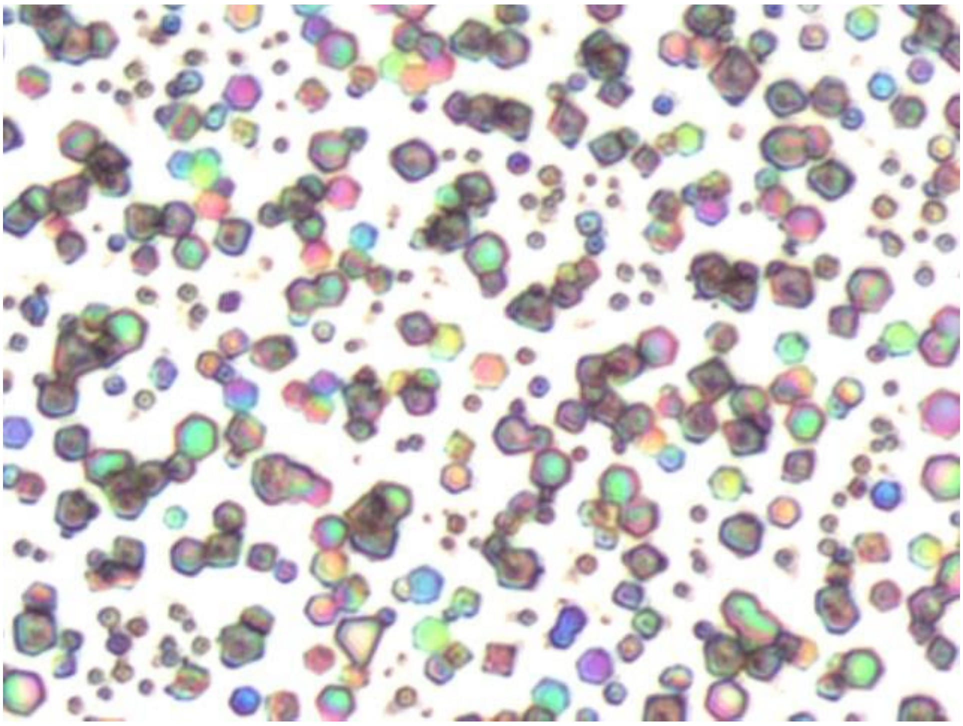

Figure S11. Optical image shows hexagonal shaped crystals of oriented **CC3** on silicon wafer substrate for the AFM analysis.

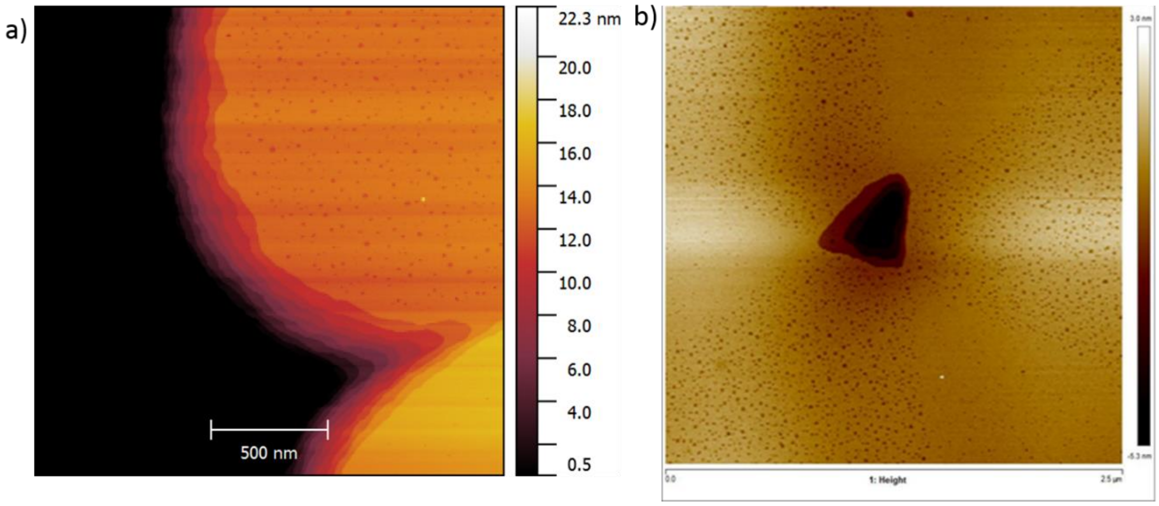

Figure S12. AFM images of additional **CC3** crystals with defects. (a) An AFM image of the edge of a quickly grown oriented CC3 crystal. A portion of two segments of the hexagonal crystal can be seen; the larger segment (at the top of the image) shows porous areas whereas the adjacent smaller lobe (beneath) has fewer defects. This shows that the localised additional pores extend all the way from the centre to the boundaries of the crystal. (b) AFM image of the centre of an additional rapidly grown oriented cage crystals with a large number of vacancy-defects, showing the generality of this effect.

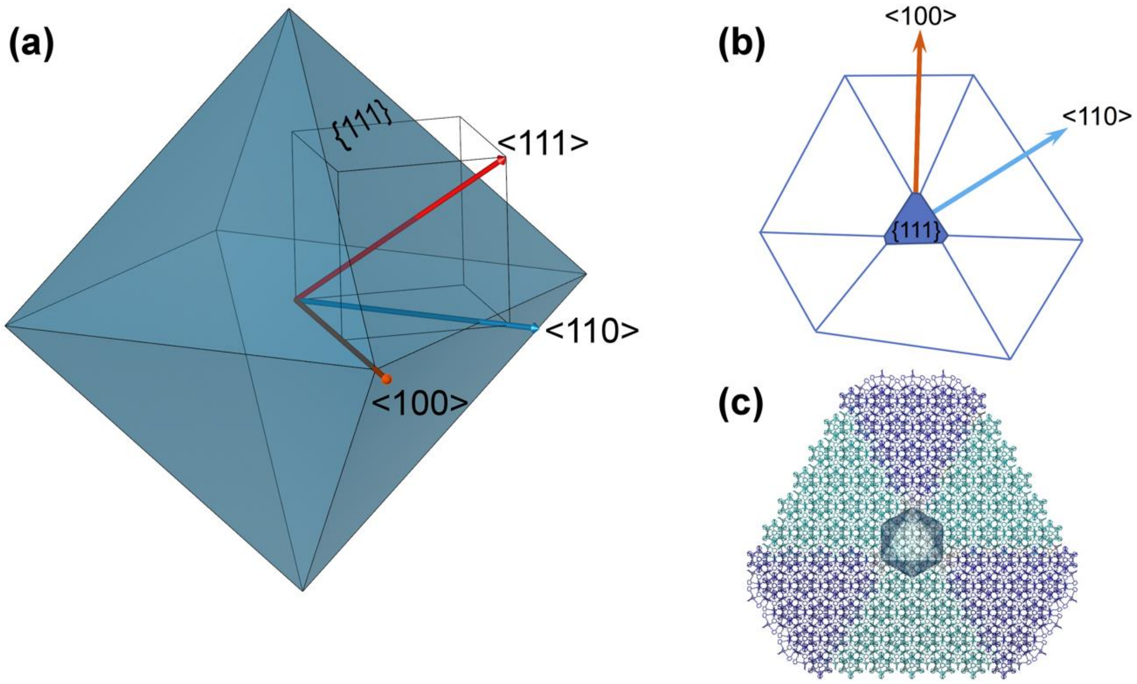

Figure S13. Schematic showing the relationship between the morphology of **CC3** crystals and the underlying cubic crystal structure. (a) Growth along the body diagonals of the cubic cell <111> relates to the formation of hexagonal layers of **CC3** molecules shown in Figure S8, which comprise the triangular {111} faces of the octahedral crystals formed by bulk **CC3**. (b) SEM (Figures 1 and S3 – S5) AFM (Figures 3 and S9 – S10) images of surface oriented **CC3** crystals show a central triangular feature surrounded by clearly defined growth sectors which form the hexagonal plate morphology. Initial formation of a triangular {111} face by growth of the crystal parallel to the surface, followed by propagation from the vertices (parallel to <100>) and edges (along <110>) would account for the observed crystal shape. (c) Differences in vacancies i.e. ‘missing cages’, and void defect concentrations between the sectors can be related to edge *vs*. point growth, with the probability of imperfections higher for growth from the edge, due to the larger area of the growth front and potential differences in the both the intermolecular interactions presented by the cages in this direction.

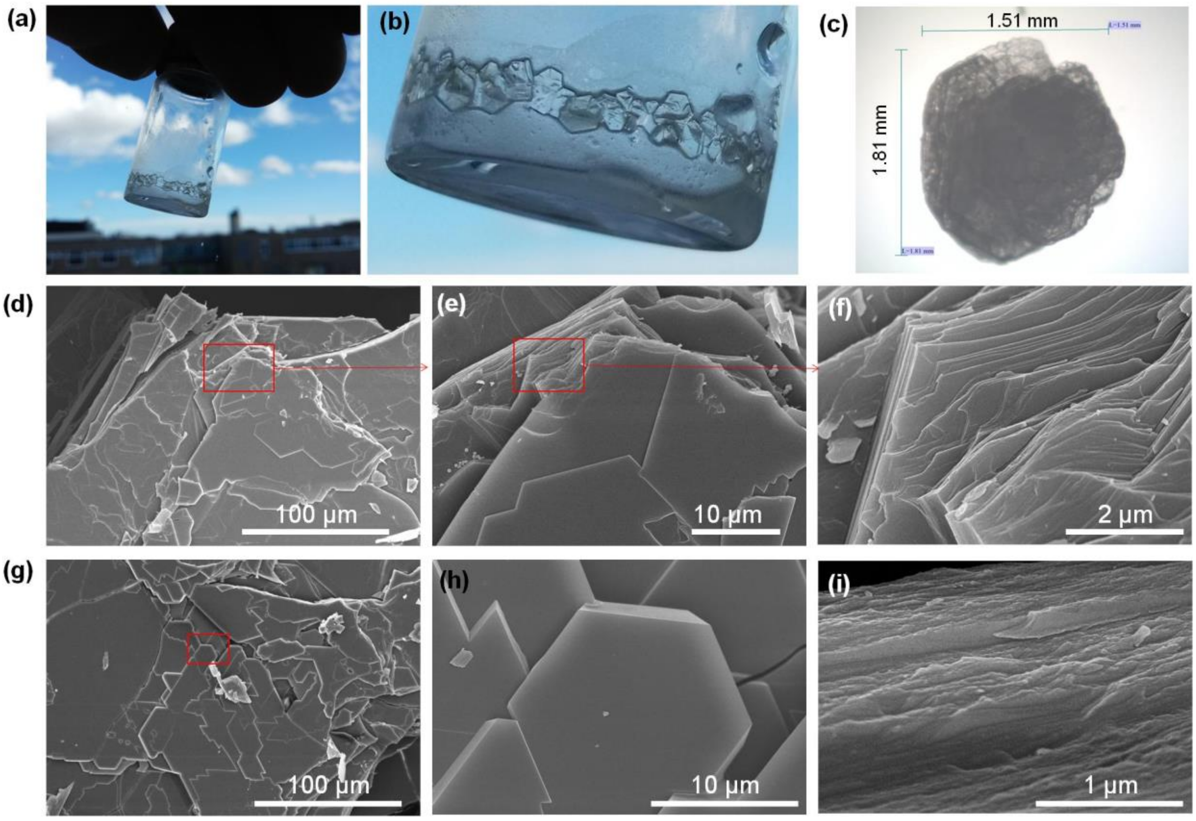

Figure S14. (a-b) Bulk oriented cage crystals grown in the glass vial. (c) Microscopy image to show the size of crystal of 1.5 x 1.8 mm. (d-i) SEM images at various magnifications and locations to show the layered structured of 2D **CC3** crystals.

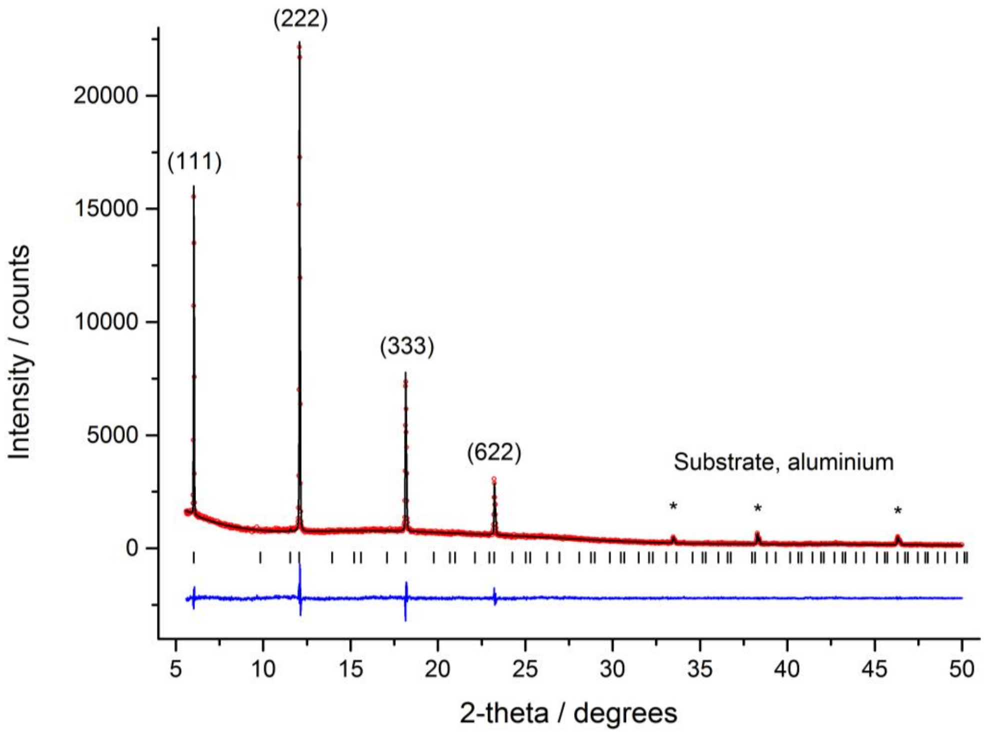

Figure S15. Final observed (red circles), calculated (black line) and difference (blue line) transmission PXRD profiles for Le Bail refinement of bulk oriented **CC3** crystals grown in the sample vial. The pattern is consistent with a **CC3α** cell (a = 25.3924(5) Å. V = 16372.3(9) Å^3; cubic $F4_132$). The reflections corresponding to diffraction from the crystallographic {111} planes dominate the pattern, indicative of the highly anisotropic 2-D plate morphology of the crystals. Diffraction intensity is observed for the (622) reflection due to the transmission geometry of the XRD experiment, in which X-rays penetrate the crystal and can be diffracted by additional planes.

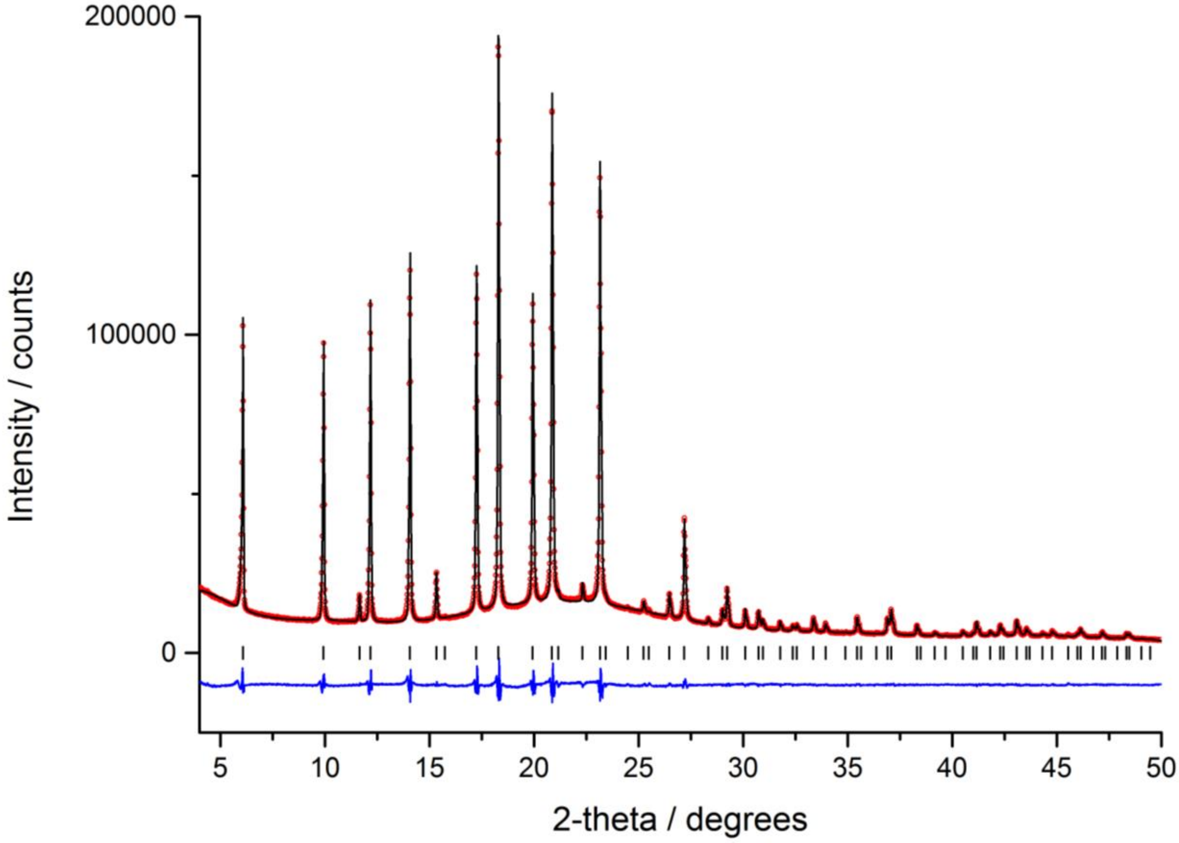

Figure S16. Final observed (red circles), calculated (black line) and difference (blue line) PXRD profiles for Le Bail refinement of bulk oriented **CC3** crystals after grinding. The refined lattice parameters (a = 25.1797(2) Å, V = 15964.3(4) Å^3; $F4_132$) are consistent with the **CC3α** structure.

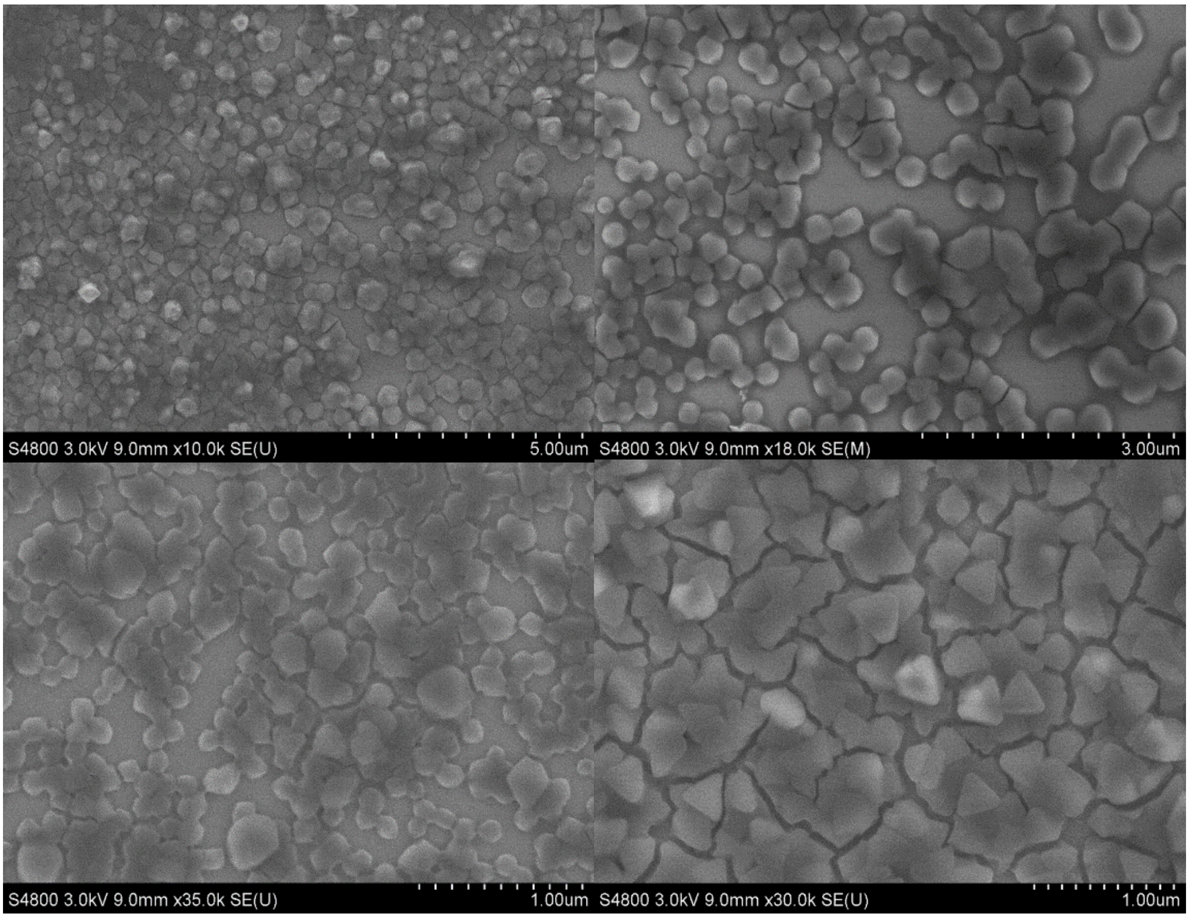

Figure S17. SEM images of oriented cage crystals on silicon wafer prepared by multiple dip coating process. After more than 100 cycles of dip coating, the surface was densely covered by cage crystals, but the top layer of crystals start to lose the orientation.

References

[1] A. A. Coelho, *TOPAS-Academic*, version 5 (Coelho Software, Brisbane, Australia, 2012), http://www.topas-academic.net.

[2] D. Nečas, P. Klapetek, *phys* **2012**, *10*, 181-188.
